# Synthesis of Dextran–Phenoxodiol and Evaluation of Its Physical Stability and Biological Activity

**DOI:** 10.3389/fbioe.2019.00183

**Published:** 2019-08-08

**Authors:** Eugene M. H. Yee, Giuseppe Cirillo, Miriam B. Brandl, David StC Black, Orazio Vittorio, Naresh Kumar

**Affiliations:** ^1^School of Chemistry, University of New South Wales, Sydney, NSW, Australia; ^2^Department of Pharmacy Health and Nutritional Sciences, University of Calabria, Rende, Italy; ^3^Lowy Cancer Research Centre, Children's Cancer Institute, University of New South Wales, Sydney, NSW, Australia; ^4^ARC Centre of Excellence in Convergent Bio-Nano Science and Technology, Australian Centre for NanoMedicine, University of New South Wales, Sydney, NSW, Australia

**Keywords:** dextran, phenoxodiol, conjugate, anti-tumor, anti-angiogenic

## Abstract

Phenoxodiol, an isoflavene anti-tumor agent, was conjugated on the polysaccharide dextran using immobilized laccase as biocatalyst. The success of the enzymatic conjugation was determined by UV-vis spectrophotometry and its functionalization degree was assessed by ^1^H NMR and was found to be 3.25 mg phenoxodiol/g of conjugate. An accelerated stability test showed that the resultant conjugate was nine times more stable than the free phenoxodiol when tested for its residual anti-oxidant activity with the Folin–Ciocalteu assay. The *in vitro* anti-proliferative activity of the conjugate was evaluated against neuroblastoma SKN-BE(2)C, triple-negative breast cancer MDA-MB-231, and glioblastoma U87 cancer cells. The conjugate was shown to be generally more potent than phenoxodiol against all three cell types tested. Additionally, the cytotoxicity and anti-angiogenic activity of the conjugate were also evaluated against non-malignant human lung fibroblast MRC-5 and human microvascular endothelial cells HMEC-1, respectively. The conjugate was found to be 1.5 times less toxic than phenoxodiol while mostly retaining 62% of its anti-angiogenic activity in the conjugate form. This study provides further evidence that the conjugation of natural product-derived drugs onto polysaccharide molecules such as dextran can lead to better stability and enhanced biological activity of the conjugate compared to the free drug alone.

## Introduction

Naturally occurring compounds or natural products, usually found in plant sources (Newman and Cragg, 2007; Newman, 2008), possess multiple bioactive properties such as anti-cancer (Barnes et al., 1996; Fotsis et al., 1997; Nijveldt et al., 2001; Ren et al., 2003; Moon et al., 2006), anti-inflammatory (Serafini et al., 2010; Kumar and Pandey, 2013), and anti-viral (Friedman, 2007; Kumar and Pandey, 2013). Flavonoids, also known as phytoestrogens, are one of the classes of natural products that are commonly found in tea and soy (Barnes et al., 1996; Cook and Samman, 1996; Kumar and Pandey, 2013).

Genistein is an isoflavone that had been extensively investigated due to its moderate anti-tumor activity (Castro and Altenberg, 1997; Zava and Duwe, 1997; Hämäläinen et al., 2007). The promising anti-cancer properties of genistein inspired the development of a structurally related isoflavene analog, phenoxodiol **1** (Alvero et al., 2006; Choueiri et al., 2006), exhibiting potent anti-tumor and anti-angiogenic activity with little toxicity toward non-malignant normal cells *in vitro* and in mouse models of human cancer (Constantinou and Husband, 2002; Kamsteeg et al., 2003; Alvero et al., 2006; Choueiri et al., 2006; Gamble et al., 2006; Silasi et al., 2009; Aguero et al., 2010). Phenoxodiol **1** has been reported to inhibit tyrosine kinases (Sapi et al., 2004), topoisomerase II (Constantinou and Husband, 2002), and the X-linked inhibitor of apoptosis as its anti-tumor mechanism (Kluger et al., 2007). These promising *in vitro* and *in vivo* results led to the clinical trials for phenoxodiol **1** in drug-resistant ovarian (NCT00382811) and prostate (NCT00557037) cancer.

Nevertheless, there is more potential for phenoxodiol **1** to be improved upon. To further enhance the anti-tumor property of phenoxodiol **1**, strategies consisting of molecular hybridization or the employment of a drug delivery vehicle have been carried out. The hybridization of phenoxodiol **1** with various moieties, such as a thiosemicarbazone (Yee et al., 2017a), a 1-amino-2-propanol side chain (Yee et al., 2013), and a Mannich base (Chen et al., 2015), and the encapsulation of phenoxodiol **1** into β-cyclodextrin (Yee et al., 2017c) have been reported to show superior anti-tumor activity than the free phenoxodiol **1**. In particular, the β-cyclodextrin–phenoxodiol complex showed a 5-fold improvement in specificity against the cancer cell types tested as compared to phenoxodiol **1** alone (Yee et al., 2017c). Hence, the use of a drug delivery vehicle for the enhancement of phenoxodiol **1** anti-tumor activity is promising.

The conjugation of natural products onto carbohydrate molecules has long been reported to improve the overall biological activity of conjugated molecules (Na et al., 2003; Pinhassi et al., 2009). One of our previous studies has demonstrated the effectiveness of increasing the anti-tumor activity of the flavonol catechin by conjugating it to dextran. The resulting conjugate exhibited potent anti-tumor activity against neuroblastoma (Vittorio et al., 2016) and pancreatic cancer cells (Vittorio et al., 2012). These encouraging results showed the potential of conjugating flavonoid molecules onto dextran. We now describe our investigation of the conjugation of the isoflavene phenoxodiol **1** to dextran, herein called dextran–phenoxodiol **2**, and the evaluation of its stability and *in vitro* biological activity.

## Materials and Methods

### Conjugation

Conjugation of phenoxodiol **1** to dextran was performed according to a previously developed protocol that involves the use of immobilized laccase (Vittorio et al., 2016). Dextran (250 mg, *Leuconostoc* spp., Mr ~6000, Sigma-Aldrich) was dissolved in MilliQ water (1.5 ml) and phenoxodiol **1** (7.5 mg dissolved in 0.5 ml of DMSO) was added to the reaction mixture, which was stirred for 45 min at room temperature to form a stable solution. Immobilized laccase (75 mg, 0.0345 U) was subsequently added and the reaction mixture was stirred for 24 h at 40°C. The catalysts were removed and the sample was purified by dialysis against a DMSO/water mixture (1:1 v/v). The sample was freeze-dried to obtain a vaporous solid.

### Stability Assay

Aliquots (1.0 ml) of free phenoxodiol **1** and the dextran–phenoxodiol **2** (0.05 mg/ml) were dissolved in PBS solution (10^−3^ M, pH 7.4) and incubated under UV irradiation with a high-pressure mercury lamp (HPK 125, Philips, Amsterdam, the Netherlands, 10 mW cm^−2^, wavelength 275 nm) for 7 h. At suitable time intervals, the total anti-oxidant activity was determined according to the Folin–Ciocalteu reagent procedure (Vittorio et al., 2012). Samples were diluted with water to 10.0 ml and Folin–Ciocalteu reagent (1.0 ml) and 2% Na_2_CO_3_ (3.0 ml) were added. The mixtures were mixed thoroughly and allowed to stand for 2 h with intermittent shaking. The absorbance was measured at 760 nm.

The same procedure was performed when compounds were incubated without UV irradiation and the residual anti-oxidant activity is expressed according to the following equation:

$$\begin{array}{l} Residual\;Activity\;\left(\%\right)=\frac{A_{x}}{A_{i}}\times 100\%\; \end{array}$$

where *A*_*x*_ and *A*_*i*_ are the total anti-oxidant activity of the solution measured in the presence and absence of UV irradiation, respectively.

### Cell Biology Techniques

Human neuroblastoma cell line SKN-BE(2)C, human breast cancer cell line MDA-MB-231, and human glioblastoma cell line U87 were cultured in DMEM medium (Invitrogen) supplemented with 10% FCS and 1% PSG. Human lung fibroblast cell line MRC-5 was cultured in MEM medium (Invitrogen) supplemented with 10% FCS, 1% L-glutamine, 2% sodium bicarbonate, 1% medium NEAA, and 1% sodium pyruvate. Human microvascular endothelial cell line HMEC-1 was grown in MCDB-131 medium (Invitrogen, Mount Waverley, Australia) supplemented with 10% FCS, 1% L-glutamine, 1 μg/ml hydrocortisone, and 10 ng/ml epithelial growth factor (BioScientific, Gymea, Australia). The HMEC-1 cells were cultured on 0.1% gelatin-coated culture plates for all experiments, except for the Matrigel assay. All cell lines were maintained at 37°C in 5% CO_2_ as an adherent monolayer and were passaged upon reaching confluence by standard cell culture techniques.

### Cell Viability Assays

SKN-BE(2)C, MDA-MB-231, and U87 cells were seeded at 3,000 cells per well and HMEC-1 cells were seeded at 2,250 cells per well in 96-well plates to ensure sustained exponential growth for 4 days. MRC-5 cells were seeded at 20,000 cells per well in 96-well plates to ensure full confluence (quiescence). Cells were treated with the compounds 24 h after seeding with a range of concentrations. After 72-h drug incubation, the treatment media was replaced with 10% Alamar Blue in fresh media and the cells were incubated for another 6 h. The metabolic activity was detected by spectrophotometric analysis, after the 72-h drug incubation, by assessing the absorbance of Alamar Blue as previously described (Pasquier et al., 2010). Cell viability was determined and expressed as a percentage of untreated control cells. The determination of GI_50_/IC_50_ values was performed using GraphPad Prism 6 (San Diego, CA, USA).

### Matrigel Anti-angiogenic Assay

The anti-angiogenic properties of the compounds were determined using the Matrigel™ assay. Twenty-four-well plates were coated at 4°C with 250 μl of Matrigel™ solution (1:1 dilution in cell culture medium) and were allowed to solidify at 37°C for 1 h before seeding. HMEC-1 cells were then seeded at 100,000 cells per well and allowed to adhere for 5 min before treatment was initiated. HMEC-1 cells were treated with 10 μM of compounds. Photographs were taken after 8 h using the 5 × objective of an Axiovert 200M fluorescent microscope coupled to an AxioCamMR3 camera driven by AxioVision 4.8 software (Carl Zeiss, North Ryde, Australia). The total surface area of capillary tubes formed was measured in five view fields per well using AxioVision 4.8 software and Matrigel™ Assay Recognition Software (M.A.R.S.) to quantitatively evaluate the vascular surface area of the structures formed.

### Statistical Analysis

All *in vitro* experiments were performed at least in triplicate and statistical significance was determined using the two-sided Student's *t*-test. All statistical analyses were performed using GraphPad Prism 6 (GraphPad Software, Inc.).

## Results and Discussion

### Synthesis and Characterization of Dextran–Phenoxodiol Conjugate

**Scheme 1:**
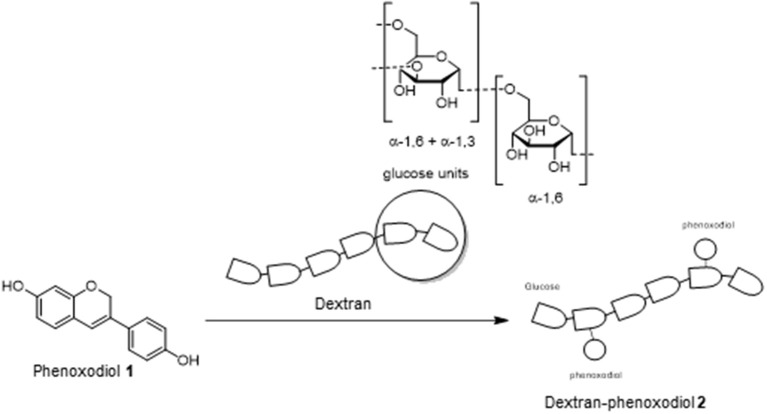
Conjugation of phenoxodiol **1** with dextran.

Laccase enzymes are commonly used as a biocatalyst in many organic reactions (Riva, 2006; Kunamneni et al., 2008). In particular, these enzymes have been investigated heavily for the preparation of polymeric polyphenols (Uyama and Kobayashi, 2003). The mechanism of action for these enzymes is through monoelectronic oxidation of the substrate (phenols on phenoxodiol **1**) to a reactive radical (Riva, 2006). The reactive intermediate could subsequently react with another substrate (dextran) to produce the polymer (Riva, 2006). These results would suggest that phenoxodiol **1** could potentially be coupled onto the dextran to produce the desired dextran–phenoxodiol **2** conjugate (Scheme 1).

To obtain the dextran–phenoxodiol **2** conjugate, an enzymatic reaction procedure that had been developed previously (Vittorio et al., 2016) was adopted and optimized. Dextran–phenoxodiol **2** was characterized through the use of UV-vis spectroscopy and the degree of functionalization was assessed by ^1^H NMR analysis (Chen et al., 2014; Vittorio et al., 2016).

**Figure 1:**
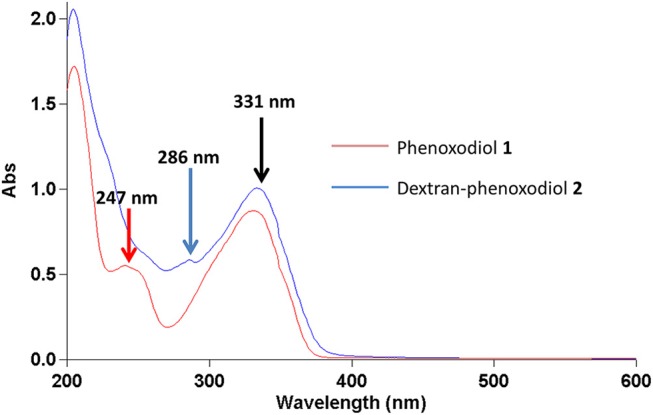
UV-vis spectrum of phenoxodiol **1** and dextran–phenoxodiol **2** in 50% aqueous MeOH.

In the UV-vis spectrum, phenoxodiol **1** exhibits two peaks at 247 and 331 nm. The 247-nm peak of phenoxodiol **1** exhibited a bathochromic shift to 286 nm after the conjugation with dextran (Figure 1). As flavonols have been shown to exhibit bathochromic shifts in UV-absorption peaks upon conjugation on their phenolic hydroxyl groups (Day et al., 2000), the spectroscopic red shift indicates that the covalent conjugation of phenoxodiol **1** onto dextran was successful.

Phenoxodiol **1** shows peaks at 247 and 331 nm while dextran–phenoxodiol **2** exhibits peaks at 286 and 331 nm.

**Figure 2:**
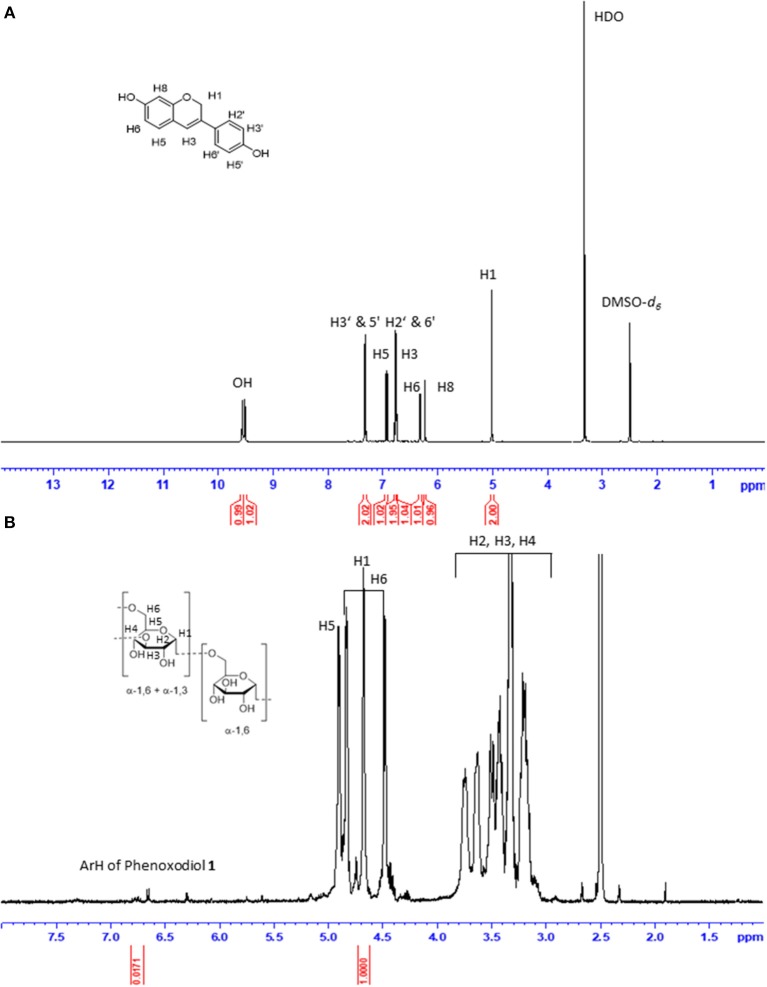
^1^H NMR spectrum of phenoxodiol **1 (A)** and 0.25 mg/ml dextran–phenoxodiol **2 (B)** in DMSO-*d*_6_.

The functionalization degree (*f*) of dextran–phenoxodiol **2** (expressed as milligrams of phenoxodiol **1** per gram of conjugate **2**) was assessed by ^1^H-NMR spectroscopy (Figure 2). The *f* value was calculated from the integration of the seven aromatic protons of phenoxodiol **1** (δ 6.70–6.80 ppm) and the anomeric protons of dextran (δ 4.65–4.75 ppm) according to the following Equation (1) (Chen et al., 2014; Vittorio et al., 2016):

(1) $$\begin{array}{r} f=\frac{\frac{1}{7}\left(\text{Integration from }6.70\text{ to }6.80\text{ ppm}\right)\cdot M_{\text{w}1}}{\text{Integration from }4.65\text{ to }4.75\text{ ppm}\cdot M_{\text{w}2}+\frac{1}{7}\;\left(\text{Integration from }6.70\text{ to }6.80\text{ ppm}\right)\cdot M_{\text{w}1}} \end{array}$$

where *M*_w1_ and *M*_w2_ are the molecular weights of phenoxodiol **1** (240.25 mol/g) and glucose residues (180.16 mol/g), respectively. Using the formula, the *f* value was calculated to be 0.00325 (3.25 mg of phenoxodiol **1** per gram of conjugate **2**). As compared to the *f* value for the enzymatic conjugation of catechin to dextran [128 mg of catechin per gram of conjugate (Vittorio et al., 2016)], the value for dextran–phenoxodiol **2** was low. The low degree of functionalization was thought to be attributed to the lower number of phenolic hydroxyl groups on phenoxodiol **1** (two) as compared to catechin (four). Regardless of the degree of functionalization, the conjugate **2** was further evaluated on its stability and biological properties.

### Stability of Phenoxodiol and Its Conjugate

**Figure 3:**
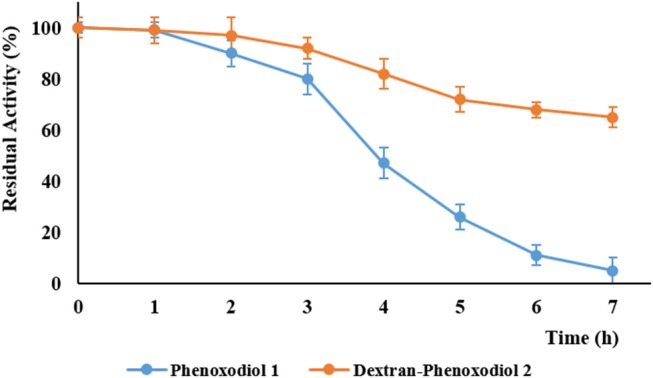
Residual activity of phenoxodiol **1** and dextran–phenoxodiol **2** after undergoing accelerated degradation conditions. *n* = 3; *bars*, SEM.

The accelerated stability testing assay is a method that is commonly used to predict the stability of pharmaceuticals (Waterman and Adami, 2005). The compounds are subjected to UV radiation at 350 nm, which simulates the environment of a compound being exposed to light over a long period of time. This allows the prediction of the stability of the compound within a short period. After 7 h of UV radiation, the residual activity of the compounds was determined according to the Folin–Ciocalteu method (Vittorio et al., 2012), measuring the total anti-oxidant activity. Phenoxodiol **1** showed 7 (±4) % residual activity, while dextran–phenoxodiol **2** exhibited 65 (±5) % residual activity (Figure 3). This suggests that the conjugation of phenoxodiol **1** onto dextran leads to an overall 9-fold increase in stability. The resulting increase in stability of the conjugate **2** is not surprising as other dextran–drug conjugates have also shown improvement to their stability in both *in vitro* and *in vivo* settings (Mehvar, 2000; Khandare and Minko, 2006).

### Biological Activity

#### Cell Viability Assay of Phenoxodiol and Its Conjugate

To determine if the dextran–phenoxodiol **2** conjugate possesses better anti-cancer properties than free phenoxodiol **1**, the anti-proliferative activity of these compounds was investigated against three different cancer cell types and one endothelial cell line. The cancer cell lines selected are the neuroblastoma cells SKN-BE(2)C, the triple-negative breast cancer cells MDA-MB-231, and the glioblastoma cells U87, while the endothelial cell line consists of the human microvascular endothelial cells HMEC-1. Additionally, a cytotoxicity assay against the non-malignant human lung fibroblast cells MRC-5 was performed to determine the specificity of the conjugate **2**.

The anti-proliferative assay measures the growth inhibition concentration (GI_50_) for each compound against the cancer and endothelial cells while they undergo rapid cellular division during the cancer process. Compounds were tested up to 100 μM as any activity above 100 μM is deemed ineffective for treatment. The cytotoxicity assay, on the other hand, determines the concentration (IC_50_) at which the compounds are toxic to the non-dividing (quiescence) cells.

**Figure 4:**
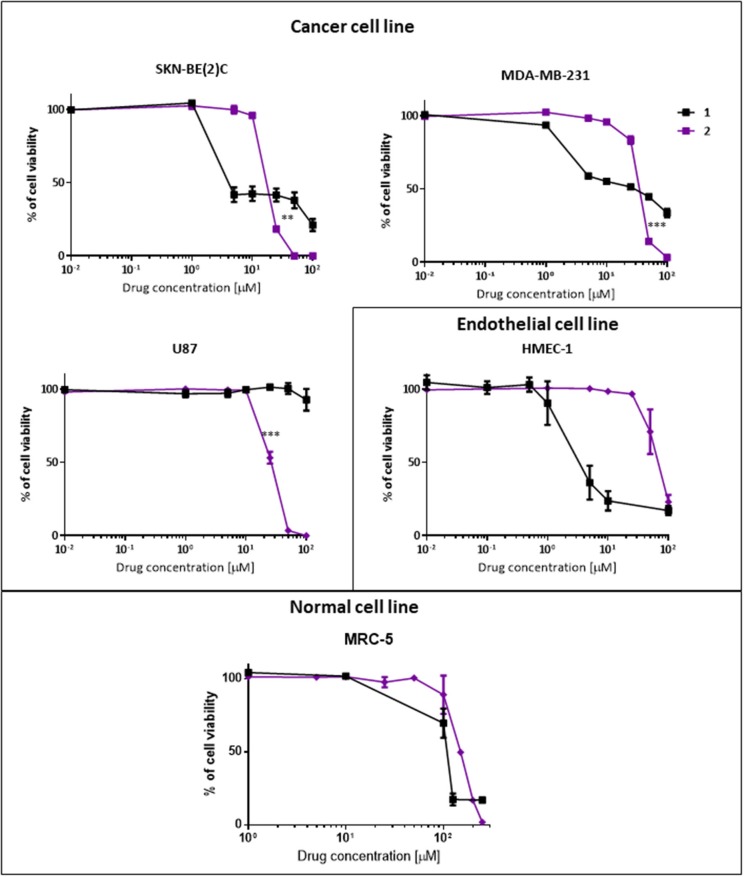
*In vitro* anti-proliferative and cytotoxicity activity of phenoxodiol **1** and dextran–phenoxodiol **2** against cancer, endothelial and non-malignant cells. Cell viability is measured on SKN-BE(2)C, MDA-MB-231, U87, HMEC-1, and MRC-5 cells using the Alamar Blue assay after 72-h incubation with a range of drug concentrations. Points as a % of cell proliferation as compared to untreated cells, *n* = 3; *bars*, SEM (**p < 0.05, ***p < 0.005).

Dextran–phenoxodiol **2** (GI_50_: 18.9 μM) was less active than phenoxodiol **1** (GI_50_: 4.5 μM) in terms of GI_50_ values against neuroblastoma cancer cells SKN-BE(2)C. However, examination of the dose–response curve of phenoxodiol **1** shows that neuroblastoma cancer cell growth was not completely inhibited even at 100 μM of drug (Figure 4), which could be due to the activation of cell rescue mechanisms induced by drug treatment (Moschovi et al., 2015). In contrast, dextran–phenoxodiol **2** was able to fully inhibit neuroblastoma growth at concentrations of 50 μM or above. Furthermore, at 25 μM, dextran–phenoxodiol **2** was already significantly more potent than phenoxodiol **1** (*p* < 0.05).

When tested on the triple-negative breast cancer cells MDA-MB-231, phenoxodiol **1** (GI_50_: 31.3 μM) and dextran–phenoxodiol **2** (GI_50_: 37.0 μM) exhibited similar potency. However, similar to the neuroblastoma cancer cells, dextran–phenoxodiol **2** is significantly more active than phenoxodiol **1** (*p* < 0.005), and could achieve full inhibition of breast cancer cell growth at higher dosages of 50 μM and above.

Phenoxodiol **1** (GI_50_: >100 μM) showed no activity at the highest dose tested against glioblastoma cells U87. Significantly, dextran–phenoxodiol **2** (GI_50_: 26.7 μM) exhibited moderate activity (*p* < 0.005). The higher anti-proliferative activity of dextran–phenoxodiol **2** compared to phenoxodiol **1** alone could possibly be due to the enhanced uptake of the drug into cancer cells as a result of its conjugation onto the sugar molecule (Macheda et al., 2005). Furthermore, various dextran–drug conjugates have shown an increased accumulation of the conjugates in the cancer cells as compared to the individual drug compound (Takakura et al., 1984; Mitra et al., 2001; Nagahama et al., 2015).

In addition, dextran–phenoxodiol **2** was tested against the endothelial cells HMEC-1, which are used in the anti-angiogenic assay. Dextran–phenoxodiol **2** (GI_50_: 68.5 μM) exhibited lower potency than phenoxodiol **1** (GI_50_: 4.1 μM) against HMEC-1 cells.

To determine if any improvement in anti-proliferative activity of dextran–phenoxodiol **2** in cancer cells was associated with higher toxicity to normal cells, the cytotoxicity of dextran–phenoxodiol **2** was investigated against the non-malignant human lung fibroblast cells MRC-5. The data revealed that dextran–phenoxodiol **2** (IC_50_: 150 μM) was 1.5 times less toxic than phenoxodiol **1** (IC_50_: 108 μM) against MRC-5 cells (Table 1).

**Table 1:** GI_50_/IC_50_ and specificity values of phenoxodiol 1 and dextran–phenoxodiol 2 against different cancer cell lines.

**Cell line**	**GI**_****50****_**/IC**_****50****_ **(μM)**	
	**Phenoxodiol 1**	**Dextran–Phenoxodiol 2**
SKN-BE(2)C	4.5 ± 0.5	18.9 ± 0.1
MDA-MB-231	31.3 ± 7.0	37.0 ± 7.0
U87	>100	26.7 ± 2.8
HMEC-1	4.1 ± 0.6	68.5 ± 11.5
MRC-5	108 ± 3.1	150 ± 1.2
**SPECIFICITY**		
SKN-BE(2)C	24.0	7.9
MDA-MB-231	3.5	4.1
U87	<1	5.6
HMEC-1	26.3	2.2

Subsequently, the specificity of the compounds was calculated using the following Equation (2):

(2) $$\begin{array}{r} \text{Specificity}=\frac{\text{I}{\text{C}}_{50}\text{ of compounds against non}-\text{malignant }\mathrm{cell}\;\mathrm{line}}{\text{G}{\text{I}}_{50}\text{ of compound against cell line of interest}} \end{array}$$

A specificity value higher than 1 indicates that the compound is more active against cancer cells than normal cells, with higher values representing higher selectivity. Compounds with a value of <1 are considered to be non-specific.

The conjugate **2** showed good overall specificity ratios between 4.1 and 7.9 for the three cancer cell lines (Table 1). Dextran–phenoxodiol **2** had better specificity values for MDA-MB-231 (4.1) and U87 (5.6) than the free phenoxodiol **1** (MDA-MB-231: 3.5; U87: <1). However, the conjugate **2** showed lower specificity values for SKN-BE(2)C (7.9) as compared to phenoxodiol **1** (24.0). Nevertheless, any values above 1 would indicate that the compound had a higher specificity toward the cell line of interest than the non-malignant cell line. Furthermore, as described earlier, dextran–phenoxodiol **2** would require a lower dose than phenoxodiol **1** to fully inhibit the growth of the neuroblastoma and breast cancer cells. This total inhibition of the cancer cell growth is highly desirable as this will reduce the chances of the cancer cells developing resistance to phenoxodiol **1** (Housman et al., 2014).

#### Anti-angiogenic Activity of Phenoxodiol and Its Conjugate

Angiogenesis, the formation of new blood vessels, is crucial to the development of solid tumors. By providing cancer cells with nutrients and oxygen, tumors are able to grow and metastasize (Carmeliet and Jain, 2000; Potente et al., 2011). Hence, the ability to inhibit angiogenesis is a highly desirable property for an anti-tumor agent (Yee et al., 2017b). To determine if dextran–phenoxodiol **2** had improved anti-angiogenic activity over the free phenoxodiol **1**, the Matrigel assay was carried out. In this assay, the surface area of the vascular structure formed by the endothelial cells after an incubation period of 8 h was used as an indication of angiogenic activity.

**Figure 5:**
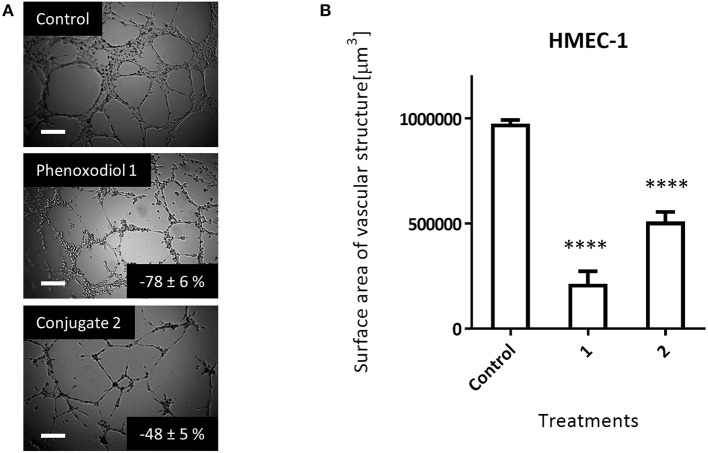
Effect of 10 μM phenoxodiol **1** and dextran–phenoxodiol **2** treatment on HMEC-1 angiogenic activity assessed with the Matrigel™ assays. **(A)** Representative photographs of HMEC-1 cells following drug treatment. *Scale bar*, 200 μm. **(B)** Total surface area of vascular structure following drug treatment. *n* = 3; *bars*, SEM (****p < 0.0001).

At 10 μM, phenoxodiol **1** showed a potent anti-angiogenic effect, inhibiting 78 (±6) % of the vascular structure formed as compared to the control (Figure 5). Meanwhile, 10 μM of dextran–phenoxodiol **2** inhibited 48 (±5) % of the vascular structure formed, indicating that it retained 62% of the anti-angiogenic activity of phenoxodiol **1** at the same concentration.

Notably, as dextran–phenoxodiol **2** (GI_50_: 68.5 μM) was about 17 times less toxic to HMEC-1 cells than phenoxodiol **1** (GI_50_: 4.1 μM) in the anti-proliferative assay, this suggests that the conjugate **2** might show even better anti-angiogenic activity compared to phenoxodiol **1** at an equitoxic dosage of drug. The improvement to anti-angiogenic activity of the overall conjugate has also been shown by other polymer–drug conjugates (Duncan, 2006; Miller et al., 2011).

## Conclusion

The conjugation of phenoxodiol **1** to dextran was successfully achieved using immobilized laccase, resulting in the generation of dextran–phenoxodiol **2**. Dextran–phenoxodiol **2** showed better stability than the free phenoxodiol **1** under the accelerated stability test. Moreover, dextran–phenoxodiol **2** showed improvement to specificity for its *in vitro* anti-proliferative activity than phenoxodiol **1** across two different cancer cell lines. Importantly, dose–response data suggested that unlike phenoxodiol **1**, the conjugate **2** could potentially prevent the induction of cell rescue mechanisms in SKN-BE(2)C and MDA-MB-231 cancer cells. Furthermore, the conjugate **2** exhibited activity (26.7 μM) against U87 brain cancer cells, whereas phenoxodiol **1** had no activity even at a high dose of 100 μM. Dextran–phenoxodiol **2** also showed lower toxicity to the non-malignant human cell line compared to phenoxodiol **1**, while still retaining most of the anti-angiogenic activity of the free drug. This study provides further evidence that the conjugation of natural product-derived drugs onto polysaccharide molecules such as dextran can lead to better stability and enhanced biological activity of the conjugate compared to the free drug alone.

## Data Availability

All datasets generated for this study are included in the manuscript/supplementary files.

## Author Contributions

EY contributed to the conception and design, acquisition of data, analysis and interpretation of data, statistical analysis, and writing of the manuscript. GC contributed to the conception and design, acquisition of data, analysis and interpretation of data, statistical analysis, and proofreading of the manuscript. MB and DB contributed to the technical assistance and proofreading of the manuscript. NK and OV obtained funding and contributed to the conception and design, acquisition of data, analysis and interpretation of data, study supervision, and revision of the manuscript. All authors discussed the results and commented on the manuscript.

### Conflict of Interest Statement

The authors declare that the research was conducted in the absence of any commercial or financial relationships that could be construed as a potential conflict of interest.
